# Ceftriaxone-resistant *Salmonella enterica* serotype Typhi in a pregnant traveller returning from Karachi, Pakistan to Denmark, 2019

**DOI:** 10.2807/1560-7917.ES.2019.24.21.1900289

**Published:** 2019-05-23

**Authors:** Anne Line Engsbro, Hans Søe Riis Jespersen, Maria Ingeborg Goldschmidt, Sarah Mollerup, Peder Worning, Martin Schou Pedersen, Henrik Westh, Uffe Vest Schneider

**Affiliations:** 1 Department of Clinical Microbiology, Copenhagen University Hospital Hvidovre, Hvidovre, Denmark; 2 Gastrounit, Medical division, Copenhagen University Hospital Hvidovre, Hvidovre, Denmark; 3 Department of Infectious Diseases, Copenhagen University Hospital Hvidovre, Hvidovre, Denmark; 4 University of Copenhagen, Copenhagen, Denmark

**Keywords:** typhoid fever, meropenem, azithromycin, XDR, carbapenemase-producing, pregnancy

## Abstract

We describe a ceftriaxone-resistant *Salmonella* Typhi bacteraemia in a pregnant woman returning from a family visit in Pakistan. Whole genome sequencing confirmed similarity to a Pakistani outbreak clone. Pregnancy and unawareness of this outbreak delayed appropriate antibiotic therapy. Concurrently, we detected faecal carriage of a carbapenemase-producing *Escherichia coli.* Awareness of the ongoing outbreak should affect empiric treatment of typhoid fever and hygiene precautions in travellers returning from Pakistan. Meropenem may be warranted in severe cases.

In Denmark, typhoid fever is a rare infection in returning travellers with fewer than 100 cases in our laboratory during the past 20 years. On a global level, the World Health Organization (WHO) estimates between 11 and 22 million cases with 128,000 to 161,000 deaths annually [1]. Since November 2016, an outbreak of *Salmonella enterica* serotype Typhi resistant to ampicillin, trimethoprim/sulfamethoxazole, chloramphenicol, fluoroquinolones and ceftriaxone has been ongoing in the Sindh Province in Pakistan, especially in Karachi and Hyderabad [2]. We here describe one imported case of ceftriaxone-resistant *S.* Typhi bacteraemia related to travel to Karachi, Pakistan and detected in Denmark in April 2019. 

## Case description

An otherwise healthy 15-week-pregnant woman in her 30s experienced fever, diarrhoea and abdominal pains while visiting relatives in Karachi, Pakistan during March and April 2019. In Pakistan, she had been treated with oral cefixime for 1 week. Upon return to Denmark, 2 weeks after initial symptoms, she presented to the emergency department but was not admitted as she was afebrile with normal pulse and blood pressure. No blood tests were done. Two days later, her family doctor admitted her to the Copenhagen University Hospital Hvidovre with fever and abdominal pain. The Figure shows the sequence of antibiotic treatment starting from admission, with corresponding daily level of C-reactive protein (CRP; normal < 10 mg/L) and body temperature.

**Figure:**
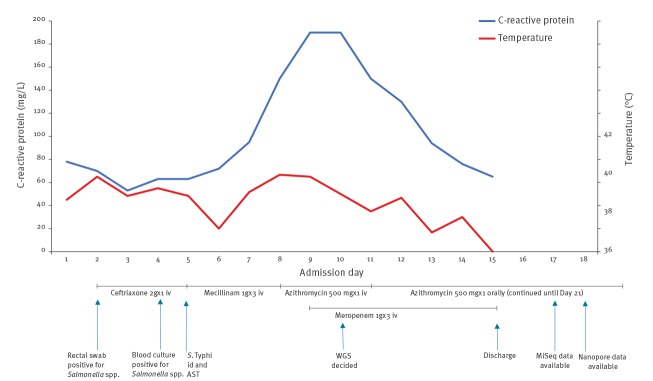
Body temperature, C-reactive protein level, antibiotic treatment and microbiological diagnostics during and after admission in a pregnant traveller returning with typhoid fever from Pakistan to Denmark, April–May 2019

AST: antibiotic susceptibility testing; id: identification; iv: intravenous; WGS: whole genome sequencing.

Antibiotic treatment and microbiological findings are listed underneath the graph.

The day after admission (Day 2), an in-house PCR on a rectal swab was positive for *Salmonella* spp. Based on this finding and because fever and tachycardia continued, treatment with intravenous (iv) ceftriaxone 2 g once daily was started. On Day 4, *Salmonella* spp. was detected by direct matrix-assisted laser desorption/ionization (MALDI Sepsityper, Bruker, Billerica, United States (US)) in blood cultures drawn on Day 2 (Bactec, BD Diagnostics, New Jersey, US). On Day 5, *S*. Typhi was identified by agglutination test (Salmonella Antisera, SSI Diagnostica, Hilleroed, Denmark). Based on the results of antibiotic susceptibility testing (AST), reported below, treatment was changed to iv mecillinam (1 g three times/day), a decision also taken in consideration of the pregnancy since penicillins are generally considered safe during pregnancy. The patient deteriorated on this treatment and on Day 8, treatment was changed to azithromycin (500 mg/day iv). Because of continuing fever and further increase in CRP, meropenem (1 g three times/day) was added on Day 9. Subsequently, clinical improvement with defervescence and decreasing CRP was seen. 

After finding the ceftriaxone-resistant *S*. Typhi, carriage of other multiresistant microorganisms was suspected and the patient was screened for faecal carriage of carbapenemase-producing microorganisms. After the detection of an OXA-48 carbapenemase-producing *Escherichia coli,* the patient was isolated to prevent spread. 

The patient was discharged on Day 15 with oral azithromycin. The fetus remained healthy.

## Characterisation of the *Salmonella* Typhi isolate

The AST results, determined by disc diffusion and E-test methods and interpreted according to the European Committee on Antimicrobial Susceptibility Testing (EUCAST) breakpoints (version 9.0) [3], are presented in the Table. They confirmed the ceftriaxone-resistant phenotype of the isolate.

**Table:** Antibiotic resistance pattern in a ceftriaxone-resistant *Salmonella enterica* serotype Typhi detected in Denmark, 2019

Antibiotic	Phenotype			Genotype
	Zone diameter (mm)	MIC (mg/L)	Interpretation S-I-R^a^	Resistance gene or point mutation detected (location on the chromosome (c) and/or the plasmid (p))
Mecillinam	26	NT	S	None
Ampicillin	6	NT	R	blaTEM-1B (c + p)
Amoxicillin/clavulanic acid	19	NT	S	None
Piperacillin/tazobactam	22	NT	S	None
Cefpodoxime	6	NT	R	blaCTX-M-15 (p)
Ceftriaxone	NT	> 32	R	blaCTX-M-15 (p)
Ceftolozane/tazobactam	NT	1.0	S	None
Ceftazidime/avibactam	NT	0.25	S	None
Meropenem	32	NT	S	None
Gentamicin	24	NT	S	None
Aminoglycosides other than gentamicin	NT	NT	NT	aph[3]-1b^b^ (c + p) aph[6]-1d^c^ (c + p) aac[6]-1aa (c)
Ciprofloxacin	20	NT	R	gyrA S83F (c) qnrS1 (p)
Azithromycin	NT	2	S	None
Trimetoprim/sulfamethoxazole	6	NT	R	sul1 (c) sul2 (c + p) dfrA7 (c)
Aztreonam	NT	> 256	R	blaCTX-M-15 (p)
Tigecycline	NT	0.25	S	None
Colistin	NT	0.125	S	None
Chloramphenicol	NT	NT	NT	catA1 (c)

c: chromosome; I: intermediate sensitivity; MIC: minimum inhibitory concentration; NT: not tested; p: plasmid; R: resistant; S: sensitive.

^a^ According to European Committee on Antimicrobial Susceptibility Testing (EUCAST) breakpoints (version 9.0) [3].

^b^ Also called strA.

^c^ Also called strB.

By MiSeq (Illumina) and MinION (Oxford Nanopore Technologies, Oxford, United Kingdom (UK)) sequencing, we assembled and closed the genome (BioProject Accession PRJNA543969) and compared it to the H58 ceftriaxone-resistant *S.* Typhi reported from Pakistan (22420_1_10_Pak600006_2016, GenBank accession LT882486) [4]. Our isolate had a chromosome of 4,813,117 bp and a plasmid of 84,498 bp length. The chromosome carried resistance genes corresponding to the H58-associated composite transposon antimicrobial resistance (AMR) cassette conferring resistance to the older first-line treatments ampicillin, sulfamethoxazole/trimethoprim and chloramphenicol (multidrug-resistant phenotype) and reduced susceptibility of fluoroquinolones (Table). The plasmid was identical to the plasmid identified in the outbreak clone; an IncY plasmid carrying a CTX-M-15 gene and a qnrS1 gene conferring resistance to β-lactams including ceftriaxone and to ciprofloxacin, respectively. Resistance genes for aminoglycosides other than gentamicin were carried on both chromosome and plasmid. A single nucleotide polymorphism (SNP) analysis showed 0 SNPs difference to the Pakistan outbreak clone. We used the Northern Arizona SNP pipeline (NASP) [5] with *Salmonella enterica* serovar Typhi CT18 [6] as the reference genome for the SNP analysis.

In a faecal swab, we identified carriage of an OXA-48 carbapenemase-producing *E. coli* using the GeneXpert Xpert Carba-R (Cepheid, Sunnyvale, US) and an overnight broth with added meropenem in a concentration of 0.25 mg/L with subsequent plating on a chromID CARBA-SMART agar (bioMérieux, Marcy l’Etoile, France).

## Discussion

An outbreak of extended-spectrum β-lactamase producing (ESBL), ciprofloxacin-resistant *S.* Typhi is ongoing in Pakistan. Almost 7,000 cases have been reported in the outbreak [7], and one third of all *S.* Typhi isolates from blood cultures in Karachi are currently ceftriaxone-resistant [8]. Only one case of ceftriaxone-resistant S. Typhi related to travel to Pakistan has previously been described in the UK [4] and recently five cases in children visiting relatives in Pakistan were reported from the US [9]. Since quinolone resistance increased, ceftriaxone has been the recommended treatment of suspected typhoid fever in the returning traveller [10]. The occurrence of travel-related cases of ceftriaxone-resistant typhoid fever in Europe emphasises the need to reconsider our empirical choice of antibiotics for suspected typhoid fever for patients returning from Pakistan.

Our patient deteriorated on mecillinam treatment although the isolate was susceptible to this drug. Poor clinical response despite in vitro susceptibility has previously been described for mecillinam [11-13]. Azithromycin with the addition of meropenem cured our patient. The effect of azithromycin on S. Typhi is well documented and comparable to quinolones [14], and azithromycin can be used in pregnancy if necessary. Addition of meropenem rather than piperacillin/tazobactam was chosen because carbapenems are superior in other ESBL-producing *Enterobacterales* [15]. The carriage of a carbapenemase-producing *E. coli* was not considered when deciding treatment strategy. Our strategy was informed by the local strategy in Pakistan, where ceftriaxone-resistant *S*. Typhi is treated with azithromycin and carbapenems [2]. Our isolate was in vitro sensitive to the newer drugs ceftolozane/tazobactam and ceftazidime/avibactam, which may represent alternative treatment options if carbapenem resistance dissipates to *S.* Typhi. In general, there was consistency between AST and resistance genes identified in the isolate. For chloramphenicol and aminoglycosides, AST was not performed because these tests are not available at our laboratory.

The only case–control study on typhoid fever in pregnancy is from Pakistan and found no increase in negative pregnancy outcomes [16]. In pregnant women, the mother should be the main focus of treatment and choice of antibiotic should follow the above recommendations. Since fluoroquinolones are contraindicated in the second and third trimester of pregnancy, azithromycin is currently the only available oral treatment for carriers of the ceftriaxone-resistant *S.* Typhi during pregnancy. Third- and newer generation cephalosporins and meropenem are discouraged in pregnancy because of lack of data. However, in our case, clinical improvement coincided with the addition of meropenem to the azithromycin treatment. The fetus was followed with ultrasound scans and was found to be healthy. 

The plasmid carried by the ceftriaxone-resistant *S.* Typhi has previously been described in *E. coli* from four different continents [4]. The clustering of cases around sewage lines in Hyderabad, Pakistan, suggests that the outbreak can be attributed to drinking water contaminated by sewage [2]. Faecal carriage of resistance genes is prevalent in travellers from South Asia as illustrated by identification of resistance genes in toilet waste from airplanes [17]. Finding a multidrug-resistant *S.* Typhi in a returning traveller indicates exposure to faecally contaminated water or foods, and the clinician must suspect the presence of other multi-resistant gastrointestinal bacteria; as was the case in our patient. Screening for multi-resistant *Enterobacterales* and hygiene precautions should therefore be considered in hospitalised returning travellers.

## Conclusion

Ceftriaxone-resistant *S.* Typhi is here described in an imported European case. Ceftriaxone resistance needs to be taken into consideration when choosing empirical treatment for patients returning from Pakistan with suspected typhoid fever. Azithromycin and meropenem had clinical effect, while the patient deteriorated on mecillinam. Isolating a multidrug-resistant *S*. Typhi should raise the concern of carriage of other multi-resistant microorganisms transmitted by the faecal-oral route.
